# A comparative study on the regulatory region of the *PERIOD1* gene among diurnal/nocturnal primates

**DOI:** 10.1186/s40101-016-0111-9

**Published:** 2016-09-28

**Authors:** Takafumi Katsumura, Yukiko Fukuyo, Shoji Kawamura, Hiroki Oota

**Affiliations:** 1 Department of Anatomy, Kitasato University School of Medicine, 1-15-1 Kitasato, Minami-ku, Sagamihara, Kanagawa 252-0374 Japan; 2 Department of Integrated Biosciences, Graduate School of Frontier Sciences, University of Tokyo, 5-1-5 Kashiwanoha, Kashiwa, Chiba 277-8562 Japan

**Keywords:** Diurnal, Nocturnal, Circadian clock, *PERIOD1*, GC content

## Abstract

**Background:**

The circadian clock is set up around a 24-h period in humans who are awake in the daytime and sleep in the nighttime, accompanied with physiological and metabolic rhythms. Most haplorhine primates, including humans, are diurnal, while most “primitive” strepsirrhine primates are nocturnal, suggesting primates have evolved from nocturnal to diurnal habits. The mechanisms of physiological changes causing the habits and of genetic changes causing the physiological changes are, however, unknown. To reveal these mechanisms, we focus on the nucleotide sequences of the regulatory region of the *PERIOD1* (*PER1*) gene that is known as one of the key elements of the circadian clock in mammalians.

**Methods:**

We determined nucleotide sequences of the regulatory region of *PER1* concerning the gene expression for six primates and compared those with those of eight primates from the international DNA database. Based on the sequence data, we constructed a phylogenetic tree including both the diurnal/nocturnal species and investigated the guanine and cytosine (GC) content in the regulatory region.

**Results:**

The motif sequences regulating gene expression were evolutionary conservative in the primates examined. The phylogenetic tree simply showed phylogenetic relationship among the species and no branching pattern distinguishable between the diurnal and nocturnal groups. We found two cores showing a statistically significant difference between the diurnal and the nocturnal habits related to the GC contents of the regulatory region of *PER1*.

**Conclusion:**

Our results suggest the possibility that the two cores in the upstream region of *PER1* are related to the regulation of gene expression leading to behavioral differences between diurnal and nocturnal primates.

**Electronic supplementary material:**

The online version of this article (doi:10.1186/s40101-016-0111-9) contains supplementary material, which is available to authorized users.

## Background

The transition from nocturnal to diurnal habits must have been an epoch in primate evolution. It is widely accepted that mammals were originally nocturnal. Strepsirrhini is a suborder of primates that include most of the prosimians (lemurs, galagos, pottos, lorises, except for tarsiers), which are closer to ancestral types of primates. Because many strepsirrhines have nocturnal habits, it is plausible that a common ancestor of primates was also nocturnal. In contrast, another suborder, Haplorhini, which includes the tarsiers, the simians, and even *Homo sapiens* (humans), has diurnal habits except for the genus *Aotus* (owl monkeys) and the tarsiers. Thus, primates are thought to have shifted from nocturnal to diurnal habits in the evolutionary process [1].

Many phenotypic features that characterize the lineage leading to humans must have evolved as a sequel to diurnal behavior. Strepsirrhines and haplorhines generally have a dichromatic and a trichromatic vision, respectively. These are typical features because nocturnal species would not require acute color vision, which is of more use for diurnal species [2]. The apes, close relatives of humans, commonly have uniform trichromatic vision [3] which is useful in individuals with diurnal habits.

The physiological mechanism determining whether nocturnal or diurnal habits are dominant is unclear. It is likely that the circadian phase has been changed between nocturnal and diurnal species. Almost all organisms have biological clocks that generate physiological rhythms of body temperature and metabolism at 24-h cycles, and regulate behavioral rhythms of sleepfulness and wakefulness, the circadian rhythm. In mammalians, transcriptional factors CLOCK and BMAL1 form dimers, and directly and indirectly activate transcription of the *Per* and *Cry* genes through the E-box sequence (CACGTG) that is the transcriptional factor binding (TFB) motif observed in regulatory regions, located on the upstream regions of genes in many cases. The activation is inhibited by accumulation of the PER and CRY proteins, the negative transcriptional feedback (NTF) loop. The NTF loop has a 24-h period and forms a circadian clock oscillator. By the oscillation of PER and CRY, the expression levels of the other clock and clock-related genes are controlled, and physiological and metabolic process are affected [4]. Therefore, we should consider the possibility that mutations in clock/clock-related genes and/or in the regulatory region controlling their expression pattern might be the key of the dynamic transition of the circadian phase. But the genetic mechanisms generating the opposite circadian phase in the nocturnal/diurnal lineages of primates are unknown.

To find a clue to reveal the genetic mechanism of the transition from nocturnal to diurnal habits, we have focused on the *PER1* gene because the transcriptional regulation of *PER1* has been studied extensively [4]. We sequence the ~4 kb upstream region including exon 1 of the *PER1* gene, in which three E-boxes are included, for six primates (chimpanzee, marmoset, owl monkey, lemur, greater galago, and lesser galago), and compare those with the homologous region sequences for eight primates (human, chimpanzee, gorilla, orangutan, gibbon, macaque, tarsier, and bushbaby) from the international DNA database. We hypothesize that we would find a correlation between the upstream sequence data and that of the nocturnal/diurnal groups, if the expression regulation of *PER1* affects the nocturnal/diurnal habits. If the upstream sequences of *PER1* only show phylogenetic relationships, then it appears that the reversal of the daytime and nighttime in the diurnal/nocturnal primates is not related to the expression pattern of the *PER1* gene. We investigated: (1) the number of substitutions in the E-boxes, (2) the topology of the phylogenetic tree, and (3) the guanine and cytosine (GC) contents, e.g., the proportion of GC in the ~4 kb upstream region. Our data demonstrate that the GC content of the region examined is significantly different from that in the nocturnal/diurnal grouping, whereas the substitutions of the E-boxes and the phylogenetic tree are not correlated with the grouping.

## Materials and methods

### Samples

We used DNA from six species, *Pan troglodytes* (chimpanzee), *Callithrix jacchus* (marmoset), *Aotus trivirgatus* (owl monkey), *Lemur catta* (lemur), *Otolemur crassicaudatus* (greater galago), and *Galago senegalensis* (lesser galago). Genomic DNA was extracted from blood samples supplied to H.O. and S.K. through the Joint Usage/Research Center of the Primate Research Institute of Kyoto University. For the comparative analyses, we downloaded nucleotide sequences orthologous to them from the database, ENSEMBL, for eight species, *Homo sapiens* (human, GRCh37), *Pan troglodytes* (chimpanzee, CHIMP2.1.4), *Gorilla gorilla* (gorilla, gorGor3.1), *Pongo pygnaeus* (orangutan, PPYG2), *Nomascus leucogenys* (gibbon, Nleu1.0), *Macaca mulatta* (macaque, MMUL_1), *Tarsius syrichta* (tarsier, tarSyr1), and *Otolemur garnettii* (bushbaby, OtoGar3). We examined 13 species of primates, and added *Mus musculus* (mouse, GRCm38) as an outgroup (Table 1).

**Table 1:** Characteristics of used species and their nucleotide sequences

Sequence ID	Species	Group	Behavior	Length1 (bp)	Length2 (bp)	GC (%)
Human	*Homo sapiens*	Haplorhini	Diurnal	–	3307	58.5
Per1Ai	*Pan troglodytes*	Haplorhini	Diurnal	3780	3308	58.6
Chimp	*Pan troglodytes*	Haplorhini	Diurnal	–	3312	58.6
Gorilla	*Gorilla gorilla*	Haplorhini	Diurnal	–	3202	58.1
Orangutan	*Pongo pygmaeus*	Haplorhini	Diurnal	–	3311	58.9
Gibbon	*Nomascus leucogenys*	Haplorhini	Diurnal	–	3310	58.8
Macaque	*Macaca mulatta*	Haplorhini	Diurnal	–	3043	58.6
Per1Marmoset	*Callithrix jacchus*	Haplorhini	Diurnal	3596	3126	57.3
Per1Owl	*Aotus trivirgatus*	Haplorhini	Nocturnal	3642	3161	57.7
Tarsier	*Tarsius syrichta*	Haplorhini	Nocturnal	–	3113	57.4
Per1RL	*Lemur catta*	Strepsirrhini	Diurnal	3895	3282	58.8
Per1GG	*Otolemur crassicaudatus*	Strepsirrhini	Nocturnal	4028^a^	3143	54.8
Bushbaby	*Otolemur garnettii*	Strepsirrhini	Nocturnal	–	3149	54.9
Per1LG	*Galago senegalensis*	Strepsirrhini	Nocturnal	3472	3154	55.5
Mouse	*Mus musculus*		Nocturnal	–	3014	53.9

Length1 means the length of nucleotide sequence determined by primer walking. Length2 is the length excluding the uncertain regions after a multiple alignment using MAFFT and TrimAl with a “gappyout” option

^a^The sequence that includes a gap of unknown length (see also DDBJ database: Accession number LC121029)

### PCR and sequencing

**Fig. 1:**
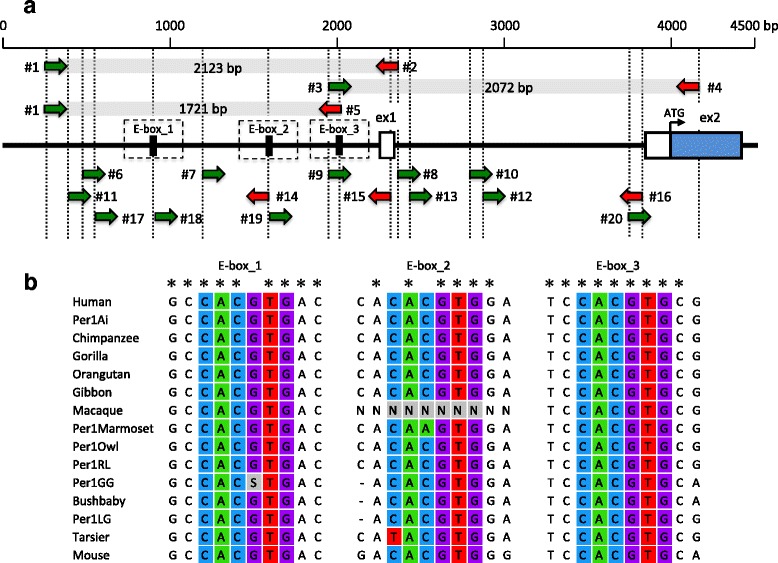
Sequence determined regulatory region **a** with the primer positions used for primer walking and **b** with three E-boxes of *PER1. Green* and *red arrows* upon the structure model of *PER1* upstream indicate the forward and reverse primers, respectively, and the *gray lines* and the *numbers* represent the lengths of the expected PCR product estimated by the human genome sequence. The numbers closest to the arrows are consistent with the primer IDs in Table 2

We divided the *Period1* promoter region into two fragments, and amplified approximately 2000 base pair (bp) fragments by polymerase chain reaction (PCR) using three pairs of primers designed based on the conserved region among primates: the primer IDs are #1–#2, #3–#4, and #1–#5 (Table 2 and Fig. 1a). The genomic DNA was used as a template for PCR in a 25 μl solution containing dNTP at 2.5 mM, 25 μM of each of primer, 0.625 U of EX Taq polymerase HS (TaKaRa Shuzo Co., Kyoto, Japan), and the reaction buffer attached to the polymerase. Reactions were carried out in a DNA Engine PTC-200 (Bio-Rad Laboratories, Inc., Hercules, USA) using the following protocol: an initial denaturing step at 95 °C for 10 min, 35 cycles of denaturation at 94 °C for 30 s, annealing at 64 °C or 69 °C (primer sets #1–2, and #2–3 and #1–5, respectively) for 30 s, extension at 72 °C for 2.5 min, and a final extension step at 72 °C for 10 min.

**Table 2:** Primer information

Purposes of use	Primer ID	Primer names	Species	Region	Length (bp)	Sequences (5′ → 3′)
PCR & Sequencing	#1	PER11stPRMF	Primates	Promoter	22	GCCCCCTCGCCTCCATTGACGG
	#2	PER11stINT1R	Primates	Intron1	24	GGGGGCAGGGAGAGGGTGGGTAGC
	#3	PER12ndPRMF	Primates	Promoter	23	GCTGGGCGGTGTCTGGGGCTCTT
	#4	PER12ndEX2R	Primates	Exon2	28	AACCATTGCTGTTGGCATCGGTGTCATC
	#5	GenPER1_1stPRM_R	Primates	Promoter	24	CTGAAGAGCCCCAGACACCGCCCA
Sequencing	#6	PER1PRM_PW1	Primates	Promoter	20	GGATCATTTTCAGGGAGAGG
	#7	PER1PRM_PW2	Primates	Promoter	21	ATATGAAGCCCTTGCCATCCC
	#8	GenPER1_PRM_PW1	Primates	Promoter	21	CGGCGGGCAGGCAGACGGAGG
	#9	PER1INT1_PW3	Primates	Intron1	20	CAGAGGGCACACCGTCGTCC
	#10	GenPER1_INT1_PW2	Primates	Intron1	21	GGCGCTAGTTCCTGCTGTTGG
	#11	GenPER11stPRM_PW1	Primates	Promoter	21	CTATAATCCTAGCACTTTGGG
	#12	PER1PRM_PW5R	Primates	Promoter	20	GGTGTCTAGTACTTCCCTCC
	#13	GenPER1_PW3R	Strepsirrhines	Exon1	20	GTCCTCCGCACGCCTGCCCG
	#14	ShoPER1_PW1	Lesser galago	Promoter	21	CCCTAGGCCGGCTGTGATGTC
	#15	PER1INT1_PW4	Primates	Intron1	20	AGGGGCTAAAGAAGGAAAGG
	#16	GenPER1_PW4R	Strepsirrhines	Intron1	20	CCCACGCTCCTAGGACCCAG
	#17	OLPER1_PW1	Strepsirrhines	Promoter	20	GACCAGCCTAAGCGAGACCC
	#18	ShoPER1_PW2	Strepsirrhines	Promoter	21	GATGGAGGGGCTCTGTAACTG
	#19	LGPER1_PRM3	Strepsirrhines	Promoter	22	TACTCACCCTCTAAACACGTGG
	#20	LemurPER1_PW1	Strepsirrhines	Promoter	20	TGTCCCTCTGTAGTCCTAGC

The column “species” means the group that has used its primer for PCR and sequencing (e.g., primer ID #13 cannot be used to sequence the upstream of haplorhines *PER1* but can be used for that of strepsirrhines)

The PCR products were diluted 20-fold and used as templates in the direct sequencing reaction (following the commercial protocol) and were then analyzed in an ABI3130 DNA sequencer (Applied Biosystems, Tokyo, Japan). We determined the 3472–4028 bp nucleotide sequences for the *PER1* promoter region by primer walking; and the primers used, #5–#20, are shown in Fig. 1a. The nucleotide sequence data determined in this study were deposited into the international DNA database DDBJ/EMBL/GenBank (accession nos. LC121024–LC121029).

### Sequence alignments and data analysis

**Fig. 2:**
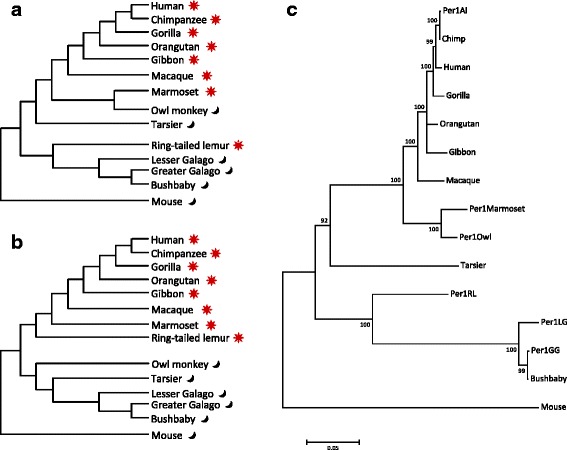
Hypothetical and actual gene trees using the upstream nucleotide sequences of *PER1*. Left **a** upper and **b** lower trees represent the putative topology that reflects only the phylogenetic relationship of the species and the clustering pattern that reflects the diurnal and the nocturnal habits, respectively. **c** Right gene tree is a maximum likelihood tree reconstructed based on 15 nucleotide sequences of the *PER1* upstream

The multiple alignments of nucleotide sequences were computed by MAFFT [5] using the EMBL-EBI web service [6] with default setting. The poorly aligned regions of the multiple alignments were removed using the program trimAl version 1.4 with a “gappyout” option [7]. Finally, after removing the poorly aligned regions, for phylogenetic analyses and calculation of GC contents, we used 3014–3312 bp (Table 1). The maximum likelihood (ML) tree was constructed based on the HKY model [8] with gamma distribution (G parameter = 1.9765) (Fig. 2c). The tree construction and estimation of the best-fit substitution model were performed using the program MEGA 6 [9]. The reliability of the tree was evaluated using 1000 bootstrap replicates. All positions containing gaps, ambiguous bases, and missing data were eliminated (complete deletion option); and then a total of 2372 positions in the final dataset was used for the tree construction.

The GC contents and distribution of GC content variation of each sequence were calculated using GC function in the “SeqinR” R package [10]: Window size and a step for the distribution of the GC contents were 500 and 25 bp, respectively. Ambiguous bases and alignment gaps contained in each window were excluded from the calculation. These statistical significances were assessed by Welch’s *t* test (*p* < 0.05). For the distribution of GC contents, *p* values were adjusted to 0.00044 after the Bonferroni corrections because of the multiple testing (Fig. 4).

## Results

### Comparison of the *PER1* regulatory regions

We determined ~4 kb sequences of the upstream region from the *PER1* exon 2 that includes three E-boxes (Fig. 1a) for six species by PCR-direct sequencing through primer walking and compared with the orthologous sequences for the nine species listed in Table 1. As we predicted, the multiple alignment showed that the E-box sequences (CACGTG) of *PER1* were well conserved in all the species (Fig. 1b). In the first E-box (E-box_1), a heterozygous site of G and C at the fourth position was detected in the greater galago (Per1GG). In the second E-box (E-box_2), substitutions from C to T at the first position and from C to A at the third position were found in the tarsier and the marmoset, respectively. No substitutions were observed in the third E-box (E-box_3). Thus, no sites distinguishable between the diurnal and nocturnal groups were found. The regions including the E-box motifs were very conservative in the primates examined in the present study.

### Reconstruction of phylogenetic tree based on *PER1* upstream sequences

We hypothesized that the branching pattern of the phylogenetic tree on the regulatory region could indicate that the nocturnal and the diurnal groups clustered separately if the habit-specific substitutions in the upstream region occurred and changed the expression patterns. The upper virtual tree represents the putative topology that reflects only the phylogenetic relationship of the species (Fig. 2a), while the lower virtual tree represents the clustering pattern that reflects the diurnal and the nocturnal habits (Fig. 2b).

To test which topology of the virtual trees is true, we constructed a phylogenetic tree based on the nucleotide sequences of the *PER1* upstream obtained from the primates (Fig. 2c). Each branch showed high bootstrap values (92–100 %), indicating that statistically, the tree topology was highly reliable. The branching pattern did not show the diurnal/nocturnal grouping, but did show the phylogenetic grouping. Thus, overall sequences of the ~4 kb upstream region were likely to have evolved reflecting the phylogenetic relationship of the species.

### Comparison of GC contents in *PER1* upstream sequences

**Fig. 3:**
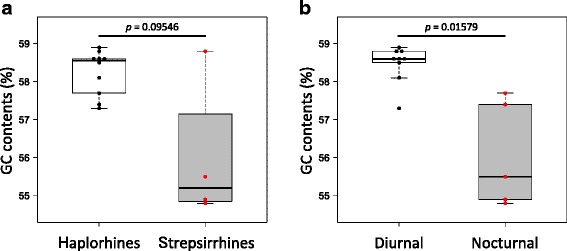
Comparisons with **a** GC contents of haplorhines and strepsirrhines and **b** nocturnal. *Boxplot* represents five-number summary statistics for each group, with *lower* and *upper error bars* indicating minimum and maximum observations excluding outliers, the *tops* and *bottoms of boxes* represent the third and the first quartiles, respectively, and the *middle bar* represents the median. *Colored dots* indicate the GC contents of the examined individual. Significant differences were identified using Welch’s *t* test

A previous study using genome-wide sequence data of the chicken showed that the GC content of the upstream regions of genes was significantly and positively correlated with expression patterns [11]. In this point of view, we surveyed overall GC contents in the *PER1* upstream sequence among the primates. The GC contents were higher in haplorhines (mean 58.3 %) than in strepsirrhines (mean 56 %). But there was no significant difference between haplorhines and strepsirrhines (*p* = 0.09546) (Fig. 3a). In contrast, we found that the GC contents of the diurnal group were significantly higher than those of the nocturnal group (*p* = 0.01579) (Fig. 3b).

**Fig. 4:**
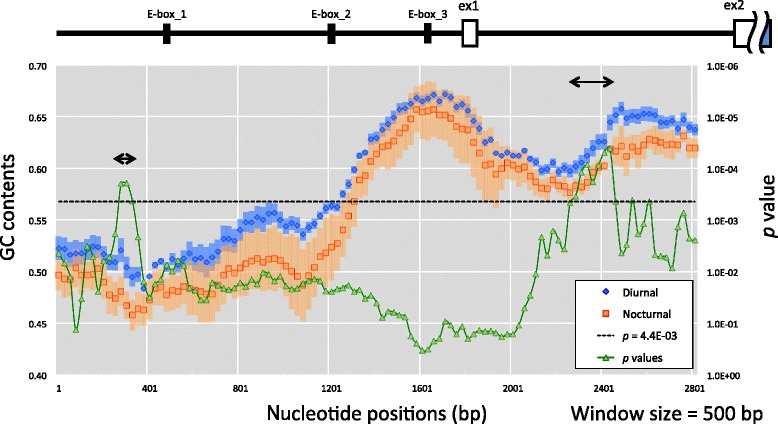
Distributions of GC contents across the upstream region of *PER1*. A double-headed arrow indicates the GC-rich region of diurnal primates compared to nocturnal ones. *Semi-transparent color* at each point represents the 95 % confidence interval of the mean

The difference of the GC contents that we detected between the diurnal and the nocturnal habits might simply represent their phylogenetic relationship because the GC contents often vary among species. To exclude the effect of phylogeny, and to specify the core regions showing a larger difference of GC contents in the upstream of *PER1*, we subsequently conducted a sliding window analysis for the diurnal and nocturnal primates (Fig. 4). The GC contents of the diurnal and nocturnal groups became higher from the distal to the proximal regions of the *PER1* gene and achieved their maximum around E-box_3 in the ~4 kb region (the blue and orange dots in the window). The two specific parts showed statistically significant differences between the diurnal and nocturnal groups (the green dots): one is the region of 223-bp upstream from the E-box_1, and the other region is in the center of intron 1. The first region showed the gap (~130 bp), which decreased the GC contents in the windows, in the sequences of the marmoset, the owl monkey, and the tarsier. This gap might contribute to the gene expression of *PER1* in the three species. The second region did not show any gaps of more than 5 bp, suggesting that the GC contents were different between the diurnal and the nocturnal habits, except for the marmoset. In addition, surprisingly the ring-tailed lemur showed a higher value of GC content across the upstream region, although the monkey was classified in Strepsirrhini (Additional file 1: Figure S1). Thus, we specified the core regions that highly contributed to the differences in the GC contents between the diurnal and the nocturnal primates.

## Discussion

We obtained the nucleotide sequences of the upstream region of the *PER1* gene for 13 species of primates and analyzed them using three different methods. First, we looked at the TFB motifs, E-boxes, in the regulatory region. Comparing the motif sequences between the primates, we found no differences between the diurnal and nocturnal species. Therefore, we suggest that the other TFB motifs might be related to the two habit styles, if the diurnal/nocturnal habits are regulated by the *PER1* gene expression patterns. Secondly, we constructed a phylogenetic tree based on the upstream sequences and found no clustering patterns distinguishable between the diurnal and nocturnal species. Thus, the primary structure of the nucleotide sequences of the regulatory region gave no details about the factors changing expression patterns of *PER1* and forming the diurnal/nocturnal habits.

There are GC-rich and GC-poor regions in vertebrate genomes, i.e., the “isochore structure” [12], and arguments that the GC content is associated with gene expression patterns [13, 14]. However, another study shows that correlations between the GC content and expression are very weak in the mammalian genome [15]. Thus, the impact of the GC content on the gene expression pattern is quite controversial in overall genome analyses. However, a more recent study, using the genome sequence data of *Gallus gallus* (chicken) downloaded from the international DNA database, separately examined protein coding sequences, complete mRNA sequences, and upstream region sequences of genes, and found that the GC content of the upstream region is significantly and positively correlated with the expression level, expression breadth, and maximum expression level [11]. In the present study, we found statistically significant differences between the diurnal and nocturnal groups in the mean GC contents of the upstream region of the *PER1* gene. It is necessary to examine the upstream regions of other genes adjacent to the *PER1* gene and the other genes located on chromosome 17 between diurnal and nocturnal primates or between Haplorhini and Strepsirrhini to confirm whether or not the difference is only observed in the *PER1* gene. Nevertheless, this result gave rise to the speculation that the difference of the expression patterns owing to the GC contents affects the diurnal/nocturnal habits.

As an exception, the GC contents of the marmoset in the two cores were as low as the nocturnal group despite the diurnal habit. We think that there are two reasons. First, this inconsistency might be caused by the phylogenetic position of the marmoset. The clade of the new world monkeys includes both of the habits. In the clade of the marmoset, the upstream region of *PER1* may not be related to the alteration from nocturnal to diurnal. Depending on the time that the primates had obtained the diurnal habit, if the marmoset’s diurnal habit occurred independently, and was different from those occurred in the old world monkeys and apes, the circadian rhythm could be regulated by the other regions. Second, in this study, we have a limit in sampling the species: the diurnal group contains more haplorhines than strepsirrhines, whereas the nocturnal group contains more strepsirrhines than haplorhines. The bias causes ambiguity in statistical tests. This is a fundamental issue: it is difficult to obtain the nocturnal Haplorhini species and the diurnal Strepsirrhini species, because the species distribution for the diurnal/nocturnal habits is already biased in nature. Nevertheless, it must be significant to test our hypothesis using additional diurnal strepsirrhines species in the near future.

We should note that the significant correlation Rao et al. (2013) showed was not based on the mammalian genome but rather based on the avian genome. Notwithstanding, that speculation suggests experimentally examining the expression level by transforming the upstream sequences of *PER1* into cells and making a comparison between the diurnal and nocturnal primates. It must be interesting to test the owl monkey and the tarsiers because both of them are classified into Haplorhini but are nocturnal. Although we have applied the candidate gene approach focusing on the *PER1* gene, there was a genome-wide study concerning transition from diurnal to nocturnal among haplorhines [16] that attempted to discover genes showing signals of positive selection between the diurnal and nocturnal species using the genomes of six primates. Our strategy, comparing the upstream region sequences, should also be taken into consideration in genome-wide analyses, including strepsirrhines in the near future, in addition to the functional analysis to determine whether or not the differences of the GC contents have some influence on the pattern of *PER1* gene expression.

## Conclusions

A statistically significant difference between the diurnal and nocturnal habits concerning the GC contents in the regulatory region of *PER1* was found. Thus, this study successfully suggests a clue toward revealing the genetic mechanisms of the transition from the nocturnal to diurnal habits in primates.

## Additional file

Additional file 1: Figure S1. Detailed distributions of GC contents across the upstream region of *PER1*. Solid and dashed lines indicate the diurnal and the nocturnal habits, respectively. Inset graphs show the regions with significant differences in Fig. 4.
