# Supplementary figures and images for: A comparative study on the regulatory region of the PERIOD1 gene among diurnal/nocturnal primates
Source: J Physiol Anthropol. 2016 Sep 28;35:21. doi: 10.1186/s40101-016-0111-9 (PMC5039903; doi:10.1186/s40101-016-0111-9)

GC contents

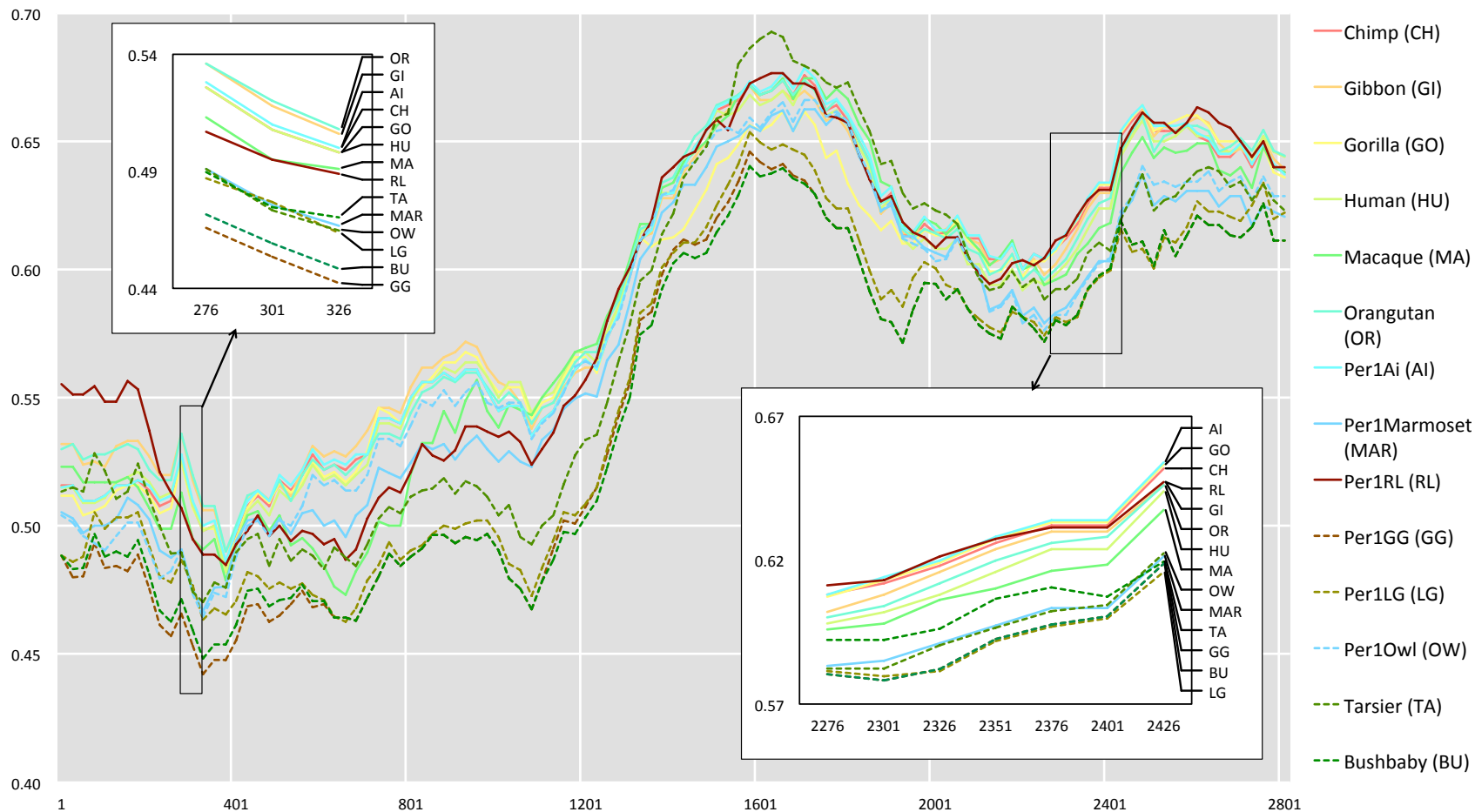

Nucleotide positions (bp)

Window size = 500 bp

Supplement: Additional file 1: Figure S1. — Detailed distributions of GC contents across the upstream region of PER1. Solid and dashed lines indicate the diurnal and the nocturnal habits, respectively. Inset graphs show the regions with significant differences in Fig. 4. [file 40101_2016_111_MOESM1_ESM.pdf]
